# Use of psychotropic drugs among workers on leave due to mental disorders

**DOI:** 10.31744/einstein_journal/2021AO5506

**Published:** 2021-05-27

**Authors:** Fabiana Vieira Garcia Leão, Alessandra Rezende Mesquita, Luciana Gonçalves de Oliveira Gotelipe, Cristiane Menezes de Pádua

**Affiliations:** 1 Departamento de Atenção à Saúde do Trabalhador Universidade Federal de Minas Gerais Belo HorizonteMG Brazil Departamento de Atenção à Saúde do Trabalhador , Universidade Federal de Minas Gerais , Belo Horizonte , MG , Brazil .; 2 Faculdade de Farmácia Universidade Federal de Minas Gerais Belo HorizonteMG Brazil Faculdade de Farmácia , Universidade Federal de Minas Gerais , Belo Horizonte , MG , Brazil .

**Keywords:** Mental disorders, Absenteeism, Psychotropic drugs, Antidepressive agents, Government Employees, Depression

## Abstract

**Objective:**

To describe the use of psychotropic drugs among civil servants with registered absenteeism due to mental disorders, and to investigate associations with duration of leave of absence.

**Methods:**

A cross-sectional study with civil servants on leave of absence due to mental disorders, between January and December 2017. Demographic, occupational and clinical variables were extracted from secondary data. Non-parametric tests were used to investigate correlations between use of psychotropic drugs and leave duration. Cluster analysis was used to investigate associations between occupational characteristics and illness profile.

**Results:**

Antidepressants were the most commonly used drugs (82.9%). Central tendency values for days on leave differed according to the number of psychotropic drugs used. In cluster analysis, a particular cluster (servants of intermediate age group and work experience – mean of 46 years and 15 years, respectively) stood out regarding use of antidepressants, severity of depression and frequency and duration of leave of absence.

**Conclusion:**

Leave of absence due to mental disorders was associated with higher rates of psychotropic drug use. The group of servants identified in this study may be a primary target for health promotion, prevention and recovery actions at the organization.

## INTRODUCTION

Mental disorders (MD) are a group of prevalent incapacitating conditions with low mortality rates. According to Global Burden of Disease (GBD) 2017, depressive and anxiety disorders are major causes of MD, particularly among individuals in the working age range. ^( 1 )^ In Brazil, these disorders are associated with a higher disease burden (35.0% and 28.0%, depressive and anxiety disorders, respectively) and are a significant contributing factor to years lived with disability. ^( 2 )^

In a Japanese study, MD were the most common cause of leave of absence for 30 days or longer among workers aged 20 to 59 years. ^( 3 )^

Mental disorders account for 15.4% of total labor costs for free-market economy due to lost labor time ^( 4 )^ (sum of hours per week), absence from work for personal reasons, or to care for sick family members (absenteeism), or due to low work performance for health-related reasons (presenteeism). ^( 4 )^

In Brazil, the prevalence of absenteeism due to MD (major cause) among public workers is approximately 25%. ^( 5 - 7 )^ However, this prevalence varies across studies according to diagnosis, occupational segments assessed and methodological aspects. ^( 8 - 10 )^ Prognostic factors or factors positively associated with MD-related absenteeism include prior MD episode, greater disease severity, previous leave of absence, comorbidity, high work burden, control and demand, female sex, low schooling level, smoking and poor self-assessed general health. Younger workers with less severe symptoms, no history of leave of absence and positive expectations regarding leave and resumption of labor activities return to work sooner. ^( 8 )^

Active, sustained therapeutic interventions for psychiatric disorders may minimize absenteeism. ^( 11 , 12 )^ Psychotropic drugs are commonly used by a large proportion of the population, ^( 13 )^ including workers. ^( 9 , 11 , 12 )^ However, studies investigating relations between the use of such drugs and absenteeism among civil servants are scarce. Most studies to date have addressed the general population or other specific subgroups. ^( 8 )^

A study with 181 workers on leave of absence for psychiatric reasons revealed relatively appropriate psychopharmacological treatment in all cases, often corresponding to polypharmacy with up to three active ingredients, the most common being antidepressants (selective and non-selective serotonin reuptake inhibitors) followed by anxiolytic drugs. ^( 11 )^ In contrast, a Swedish cohort revealed underuse of antidepressants by workers prior to and after having received temporary or permanent disability living allowance. ^( 14 )^ In a Japanese study, non-use of antidepressants was more common among workers taking longer leaves of absence due to MD. ^( 12 )^ According to the Longitudinal Study of Adult Health (ELSA Brasil), benzodiazepines and antidepressants are commonly used by university employees and lecturers. However, associations with leave of absence were not investigated in that study. ^( 15 , 16 )^

The investigation of therapeutic interventions for MD experienced by workers and associations with leave of absence duration may contribute to appropriate planning of actions aimed to promote mental health of workers and therapeutic management of these conditions.

## OBJECTIVE

To describe the use of psychotropic drugs among civil servants with registered absenteeism due to mental disorders, and to investigate associations with duration of leave of absence.

## METHODS

### Study design and experimental period

A cross-sectional study based on secondary data from federal civil servants on leave of absence due to MD, between January 1 ^st^ and December 31 ^st^ , 2017.

### Population: inclusion and exclusion criteria

The study population comprised active federal civil servants, faculty and non-faculty staff working at *Universidade Federal de Minas Gerais* (UFMG), in Belo Horizonte (MG). Inclusion criteria were as follows: at least one registered leave of absence due to MD (chapter V, group F, International Classification of Disease, 10 ^th^ Revision - ICD-10), ^( 17 )^ with medical-legal report (restricted access) in *Departamento de Atenção à Saúde do Trabalhador* (DAST) [Integrated Human Resources Administration System – Occupational Health Department] of UFMG (https://www.ufmg.br/prorh/dast/). Civil servants with registered leave of absence due to MD but no medical-legal report were excluded. Following application of exclusion criteria, the final sample comprised 202 civil servants (equivalent to 2.8% of active servants, as of March 2017).

### Data collection and variables

Data collection was conducted between July and October 2018. Data sources with restricted access were used in this study. Data were extracted from medical records of civil servants undergoing medical expert examination at DAST or from *Departamento de Administração Pessoal* (DAP) [Staff Administration Department] records. The following data were extracted from medical records: reason for leave of absence according to ICD F (major cause of leave, primary ICD, and secondary ICD, if available); number and duration (days) of leaves due to ICD F; and psychotropic and other drugs used while on leave (third and fourth levels, Anatomical Therapeutic Chemical – ATC - classification system). Demographic and labor-related variables (sex, age in June 2018, marital status, schooling level, job position, job grade, working hours at the institution and work experience were extracted from DAP records. Job grade was defined as basic (elementary education requirement), intermediate (secondary or middle level technical education requirement) or high (higher education requirement).

A pilot study was conducted to harmonize research instruments and data collection procedures. Data were entered into Excel spreadsheets (Excel, version 2010, Microsoft Corp., United States) for analysis.

### Data analysis

Data were analyzed using absolute and relative frequencies and measures of central tendency and dispersion. The Spearman correlation coefficient (rho) was used to investigate correlations between servant age and leave duration. The non-parametric Kruskal-Wallis tests was used to investigate correlations between use of psychotropic drugs and leave duration. The Mann-Whitney test was employed to analyze use of antidepressants and leave duration.

Exploratory multivariate statistical analyses (factor and cluster analysis) were used to investigate associations between servant’s professional characteristics and illness profile. Briefly, in cluster analysis, n individuals are grouped ( *i.e* ., clustered) according to known features expressed as p variables. Individuals in the same group/cluster should be similar regarding characteristics that were measured, whereas those in different groups/clusters should be heterogeneous. ^( 18 )^ Factor analysis often precedes cluster analysis and is conducted to eliminate the effect of correlations between variables. Correlated variables ( *e.g* ., work experience and age) are grouped to form new variables ( *e.g* ., functional variables), which are then used in cluster analysis. Hierarchical clustering was used to generate centroids. K-means were then used for final clustering. Kruskal-Wallis test was used to compare proportions of responses to variable categories forming clusters in cluster analysis. ^( 18 )^

Statistical analyses were conducted using the software SPSS, version 24.0.

### Ethical concerns

This research project was approved by the Ethics Committee for Research Involving Human Beings of UFMG (opinion no. 2650149, CAAE: 86430018.6.0000.5149). Participants were not interviewed and information was collected exclusively from secondary data. For this reason, this study was granter waiver of informed consent. This study was conducted in compliance with resolution 466/12, which regulates research involving human beings.

## RESULTS

### Population features

This sample comprised 202 participants. Of these 77.2% were female, 70.3% had higher level of education (undergraduate or graduate degree), 41.6% were married and approximately 70% were aged 40 years or older (mean 46.5 years; range 24.7-67.5 years) ( Table 1 ).

**Table 1:** Characteristics of servants on leave of absence due to mental disorders who used psychotropic drugs

Demographic and work-related variables	n (%)
Sex	
Female	156 (77.2)
Male	46 (22.8)
Age group, years	
20-30	12 (5.9)
30-40	44 (21.8)
40-50	66 (32.7)
50-60	65 (32.2)
60-70	15 (7.4)
Schooling level	
Elementary education	5 (2.5)
Secondary education	55 (27.2)
Higher education	40 (19.8)
Specialization course	55 (27.2)
Master’s degree	23 (11.4)
PhD degree	24 (11.9)
Marital status	
Married	84 (41.6)
Divorced	25 (12.4)
Legally separated	13 (6.4)
Single	79 (39.1)
Widow/er	1 (0.5)
Job grade*	
Basic	5 (2.5)
Intermediate	130 (64.4)
High	49 (24.3)
Lecturer	17 (8.4)
Not informed	1 (0.5)

* job grade was defined as basic (elementary education requirement), intermediate (secondary or middle level technical education requirement) or high (higher education requirement).

A large proportion of servants had a higher level of education than required for their job position: 74 out of 130 (57.0%) intermediate level servants had undergraduate or graduate degree.

Mean work experience was 15.7 years (standard deviation – SD, 9.8) and 44% of servants had been working at UFMG for more than 16 years. Mean work experience as a public server was 16.6 years (SD, 10.09).

### Leaves of absence

The number of registered leaves of absence totaled up 403, corresponding to 10,698 days (mean of 53 days/server) ( Table 2 ). Approximately 25% of servants had been on leave for 12 days or less and another 25% had been on leave for more than 70 days. Longest leave duration was 420 days. Approximately 50% of servants had been on leave a single time over the course of the experimental period.

**Table 2:** Distribuition of number of leave of absence and number of servants using psycohotropic drugs according to International Classification of Disease

ICD code grouping	Leaves of absence n (%)	Servants n (%)
F00-F09 Organic, including symptomatic, mental disorders	1 (0.2)	1 (0.5)
F10-F19 Mental and behavioral disorders due to psychoactive substance use	15 (3.7)	7 (3.5)
F20-F29 Schizophrenia, schizotypal and delusional disorders	24 (6.0)	9 (4.5)
F30-F39 Mood (affective) disorders	187 (46.4)	101 (50.0)
F40-F48 Neurotic, stress-related and somatoform disorders	154 (38.2)	108 (53.5)
F50-F59 Behavioral syndromes associated with physiological disturbances and physical factors	3 (0.7)	1 (0.5)
F60-F69 Disorders of adult personality and behavior	16 (4.0)	9 (4.5)
F70-F79 Mental retardation	1 (0.2)	1 (0.5)
Other ICD	1 (0.2)	1 (0.5)
Not informed	1 (0.2)	1 (0.5)
Total	403 (100.0)	202*

* indicates number of servants rather than sum of figures in the column, since the same servant was diagnosed with disorders from more than one ICD-10 grouping. ICD: International Classification of Diseases.

Mood (affective) disorders were the most frequent condition (46.4% of leaves of absence) and the primary diagnosis in more than half of servants. Neurotic, stress-related and somatoform disorders also stood out (38.2% of leaves of absence) and were the primary diagnosis in 53.5% of servants ( Table 2 ). These MD were also more common when secondary ICD were accounted for in making diagnosis.

### Use of drugs

The number of servants using at least one drug totaled up 187 (92.6%). More than 140 drugs were used, 14.3% of which were antidepressants, used by 82.9% of servants ( Table 3 ). “Selective and non-selective serotonin reuptake inhibitors” (N06AB), “other antidepressants” (N06AX) and “non-selective monoamine reuptake inhibitors” (N06AA) were the most commonly used antidepressive agents (62.6%, 58.1% and 9.7% of servants, respectively). “Diazepines, oxazepines, thiazepines and oxepines” (N05AH), “other antipsychotic drugs” (N05AX) and “lithium” (N05AN) were the most commonly used antipsychotics used (41.3%, 39.7% and 30.2% of servants, respectively). As to anxiolytics, “benzodiazepine derivatives” (N05BA) and “azaspirodecanedione derivatives” were used by 97.7% and 4.7% of servants, respectively.

**Table 3:** Distribution of psychotropic drugs according to the Anatomical Therapeutic Chemical classification system (third level) and number of servants on leave of absence due to mental disorders

Psychotropic drugs	Drugs n (%)	Servants n (%)
Psychostimulants, agents used for ADHD and nootropics (N06B)	1 (0.7)	1 (0.5)
Opioids (N02A)	1 (0.7)	2 (1.1)
Anxiolytics (N05B)	7 (5.0)	43 (23.0)
Psycholeptics and psychoanaleptics (N06C)	6 (4.3)	46 (24.6)
Antipsychotics (N05A)	13 (9.3)	63 (33.7)
Antiepileptics (N03A)	10 (7.1)	80 (42.8)
Antidepressants (N06A)	20 (14.3)	155 (82.9)
Other drugs (non-psychotropic drugs)	82 (58.6)	107 (57.2)
Total	140 (100)	187 (100)*

* indicates number of servants rather than sum of figures in the column, since the same server used drugs belonging to more than one pharmacological subgroup. ADHD: attention- *deficit* /hyperactivity disorder.

Median for days on leave of absence were higher (median= 30 days) among antidepressant users relative to servants that did not use these agents (median=15 days); Mann-Whitney test, p value 0.001.

A total of 86% (n=174) servants on leave of absence used psychotropic drugs of some kind, and 69% used two or more drugs simultaneously. The mean number of psychotropic drugs per server was 2.28 (SD, 1.49).

**Figure 1:**
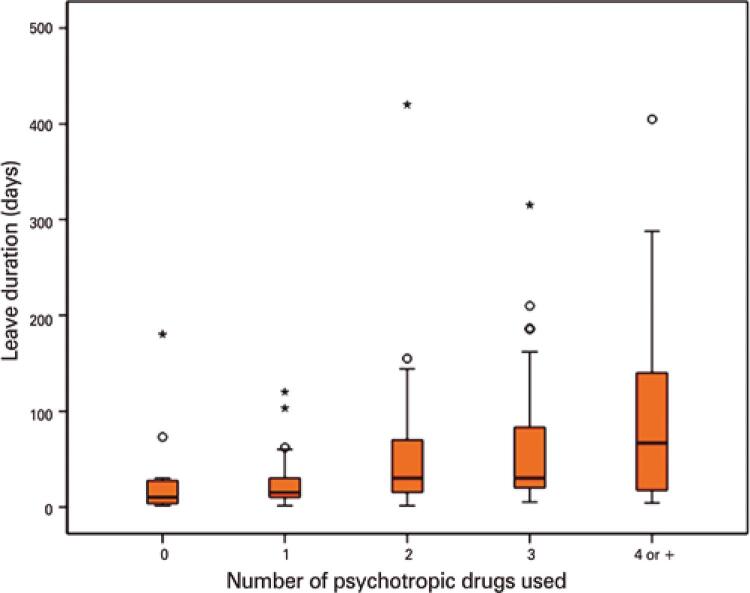
Leave of absence duration (days) according to use of psychotropic drugs

Central tendency values for days on leave differed significantly according to number of psychotropic drugs. Multiple comparisons revealed significant leave duration differences between servants who did not use psychotropic drugs and those who used two (p=0.004), three (p<0.001) and four or more (p<0.0001) drugs ( Figure 1 ).

° mild outlier; * extreme outlier.

Leave of absence duration (days) was not significantly correlated with age of servants (Spearman correlation coefficient, rho -0.026, p=0.715).

### Cluster analysis

The analysis divided data into two, three, four and five clusters. The best arrangements led to the construction of three clusters with higher R ^2^ value (0.53), which explained 53% of variability in data (α=0.05) ( Table 4 ).

**Table 4:** Characteristics of servants clusters on leave from work due to mental disorders using psychotropic drugs

Variables	Sample	Cluster			p value
		1	2	3	
Use of antidepressants	77	71	70	96	<0.001
Diagnosis of depression*	49	44	34	76	<0.001
Sex					
Female	157 (78)	59 (79)	59 (78)	39 (76)	0.08
Male	45 (22)	16 (21)	17 (22)	12 (24)	
Job grade ^†^					
Basic	5 (2.5)	4 (5)	0 (0)	1 (2)	0.33
Intermediate	130 (64)	46 (61)	51 (67)	33 (65)	
High	49 (24)	16 (21)	21 (28)	12 (24)	
Lecturer	17 (8)	9 (12)	3 (4)	5 (10)	
Marital status					
Married	84 (42)	38 (51)	32 (42)	14 (28)	0,148
Single	79 (39)	18 (24)	35 (46)	26 (51)	
Others	39 (19)	19 (25)	9 (12)	11 (22)	
Leaves of absence ^‡^	2.0±1.4	1.3±0.6	1.5±0.7	3.8±1.4	<0.001
Total of psychotropic drugs ^‡^	2.3±1.5	1.73±1.2	1.8±1.2	3.9±1.1	<0.001
Work experience at UFMG, years ^§^	15.7±9.8	24.0±6.8	8.0±4.8	15.0±9.3	<0.001
Servant age, years ^§^	46.4±9.9	54.4±5.5	38.6±6.8	46.3±9.5	<0.001
Total leave duration (days) ^‡^	53.0±67.2	25.9±24.7	28.3±30.7	129.6±91.4	<0.001

Results expressed as %, n (%) or mean±SD. * includes ICD F31, F32, F33; ^†^ job grade: basic (elementary education requirement), intermediate (secondary or middle level technical education requirement), or high (higher education requirement); ^‡^ includes “leave of absence variables”; ^§^ includes “functional variables”. UFMG: *Universidade Federal de Minas Gerais.*

Clusters 1 (n=75; 37.1%) and 2 (n=76; 37.6%) comprised servants who had been on leave for 25 to 28 days, on average, and less than twice over the course of the experimental period. Approximately 70% of servants in both clusters used antidepressants. In cluster 1, 44% of servants had diagnosis of depression, compared to 34% in cluster 2. Servants used less than two psychotropic drugs simultaneously (mean 1.7 drugs). These two clusters differed by age and work experience (mean age of 54 years and mean work experience of 24 years in cluster 1; mean age 38 years and mean work experience of 8 years in cluster 2).

Cluster 3 (n=51; 25.4%) comprised servants who had been on leave for 130 days, on average, and approximately four times in 2017. Almost all servants (96%) were on antidepressants and 76% had diagnosis of depression. Servants used, on average, 3.86 psychotropic drugs at the same time. Mean server age and mean work experience in this cluster were 46 and 15 years, respectively.

Sex, marital status and job position did not differ significantly between the three clusters.

## DISCUSSION

This study examined the use of psychotropic drugs by public servants on leave of absence due to MD and investigated associations with leave of absence duration. This duration was associated with the use of psychotropic drugs and the greater the number of drugs used, the longer the duration. This finding is in contrast with data published by other researchers, who described associations between inappropriate pharmacological treatment (nonuse of psychotropic drugs or underutilization of antidepressants) and longer leave of absence due to MD. ^( 12 )^ Associations between inappropriate pharmacological treatment and temporary or permanent disability living allowance have also been reported. ^( 14 )^

In this study, antidepressants, antiepileptics and antipsychotics were the major psychotropic drug classes used, consistent with diagnoses F30-39 (mood - affective disorders) and F40-F48 (neurotic, stress-related and somatoform disorders) recorded in approximately 85% of leaves of absence. Concurrent use of two or more psychotropic drugs, detected in a large proportion (69%) of the study population, has been defined as “psychotropic polypharmacy”. ^( 19 )^ Although monotherapy is the recommended practice, several psychiatric diseases require multiple drugs. ^( 20 )^ Polypharmacy may have been required in cases combining mood and anxiety disorders in this study, and does not necessarily indicate excessive drug use. However, findings of the ELSA-Brasil study suggested inappropriate psychotropic drug use by Brazilian university employees, with underuse of antidepressants and overuse of benzodiazepines. ^( 15 , 16 )^ Methodological and analytical differences preclude direct comparisons between this and the ELSA-Brasil study. For example, use of self-report of participants (non-faculty and faculty staff) in the ELSA-Brasil study translated into a broader investigation of psychotropic drug use (and eventually inappropriate use), whereas investigation of a relatively selected sample of individuals with medical-legal report and secondary records of use of drugs in this study may have interfered with measurement of psychotropic drug use among servants with mild to moderate MD, which are not amenable to leave of absence.

Use of antidepressants was associated with leave duration and led to division of the study population into three clusters. Clusters shared the use of antidepressants whereas magnitude of use differed, as well as some demographic and labor-related variables. Data analysis revealed a smaller group (cluster 3) of relatively young servants with intermediate work experience relative to remaining groups, which differed regarding use of antidepressants, diagnosis of depression and leave of absence frequency and duration. Concurrent use of almost four psychotropic drugs in this group apparently reflects severe depression in these servants.

Approximately 70% of servants on leave were aged 40 years or older. Similar age group of servants on leave due to MD has been reported in Alagoas ^( 21 )^ and Santa Catarina. ^( 6 )^ Another study conducted in Santa Catarina between 2010 and 2013 with 8,765 servants on leave due to MD ^( 22 )^ revealed weak correlations between servant age and leave (days) for medical treatment. Similar findings have been reported in Piauí. ^( 23 )^ In this study, groups differed by age. However, cluster 3, in which psychotropic drug (and antidepressant) use and leave frequency and duration were higher, comprised individuals of intermediate age. Likewise, a Danish cohort study revealed increased risk of leave of absence due to MD among workers on antidepressants aged 25-54 years relative to other age groups (55-64 and 18-24 years). ^( 9 )^ In other studies, leave of absence due to MD was more common among older individuals. ^( 8 , 10 , 24 , 25 )^

No differences regarding sex, marital status or job position were found in this study. Higher of MD prevalence and incidence in women in some studies ^( 8 , 10 , 24 , 25 )^ suggested depressive episodes are associated with social pressures, chronic stress e low levels of satisfaction related to traditional feminine roles, such as household chores and taking care of children. ^( 26 )^ In spite of the higher number of female servants in this sample, this profile did not seem to be a determining factor of psychotropic drug use (antidepressants). Likewise, job grade (intermediate level in most cases) was not a clustering factor in this study.

Unprecedently, this study examined the use of psychotropic drugs among civil servants and investigated associations with leave of absence due to MD at an important Brazilian higher education institution. However, some limitations must be acknowledged. Less severe cases of MD amenable to short lasting leave of absence may have been underreported, since these represent occasional events which tend to be communicated directly to higher management and may therefore not have been duly reported to the DAST. Underreporting may also occur when servants with MD do not go on leave for fear of exposure and discrimination in the work environment. Psychotropic drug use may also have been underreported in medical records. This may have resulted from incomplete reporting during expert medical assessment, memory *deficits* or embarrassment regarding the use of psychotropic drugs due to the stigma associated with MD and its treatment. Given the complexity of MD and respective pharmacological management, we also underline this study was limited to quantitative assessment of psychotropic drug use. Use of a secondary database provided limited access to information regarding the use of other drugs, and the manner in which they are used, precluding qualitative assessment of drug use appropriateness.

High rates of leave of absence among civil servants in Brazil emphasize the significance of MD in this population. ^( 5 - 7 )^ At the university evaluated in this study, mental and behavioral disorders resulted in more than 10 thousand days on leave per year, between 2011 and 2017 ^( 27 )^ and ranked highest among other causes of long lasting leave of absence. Future investigations, particularly longitudinal studies involving qualitative assessment of psychotropic drug use and potential relations with leave of absence, are needed for deeper understanding of drug use among civil servants in public higher education institutions in Brazil.

## CONCLUSION

Leave of absence due to mental disorders was associated with higher rates of psychotropic drug use. One cluster of servants stood out regarding psychotropic drug use profile. In this particular cluster, servants were of intermediate age, had intermediate work experience, took longer leaves of absence and used more psychotropic drugs (polypharmacy). This unprecedented exploratory analysis conducted with civil servants in public higher education institutions in Brazil may support the implementation of strategies related to health promotion, prevention and recovery actions related to workers.
