# Supplementary material for: Use of psychotropic drugs among workers on leave due to mental disorders
Source: Einstein (Sao Paulo). 2021 May 27;19:eAO5506. doi: 10.31744/einstein_journal/2021AO5506 (PMC8169027; doi:10.31744/einstein_journal/2021AO5506)
Supplement: Translation

# Translation: Uso de psicofármacos entre trabalhadores em afastamento laboral por transtornos mentais

**Authors:** Fabiana Vieira Garcia Leão, Alessandra Rezende Mesquita, Luciana Gonçalves de Oliveira Gotelipe, Cristiane Menezes de Pádua

**Affiliations:** 1 Brasil Departamento de Atenção à Saúde do Trabalhador, Universidade Federal de Minas Gerais, Belo Horizonte, MG, Brasil .; 2 Brasil Faculdade de Farmácia, Universidade Federal de Minas Gerais, Belo Horizonte, MG, Brasil .

**Keywords:** Transtornos mentais, Absenteísmo, Psicotrópicos, Antidepressivos, Empregados do Governo, Depressão

## Abstract

**Objetivo:**

Descrever a utilização de psicofármacos por servidores públicos com registro de absenteísmo por transtornos mentais, e analisar sua associação com a duração do afastamento do trabalho.

**Métodos:**

Estudo transversal com servidores públicos em afastamento laboral por transtornos mentais, entre janeiro a dezembro de 2017. Variáveis demográficas, ocupacionais e clínicas foram obtidas a partir de dados secundários. Foram empregados testes não paramétricos para avaliar a correlação entre o uso de psicofármacos e o tempo de afastamento. A análise de *cluster* foi utilizada para verificar a associação entre as características ocupacionais e o perfil de adoecimento do servidor.

**Resultados:**

Os antidepressivos foram os medicamentos mais utilizados (82,9%). Observaram-se diferenças nos valores centrais de dias de afastamentos por número de psicofármacos utilizados. Na análise de *cluster* , um dos conglomerados (servidores com idade e tempo de trabalhos intermediários – média 46 anos de idade e 15 anos de trabalho) destacou-se em relação à utilização de antidepressivos, gravidade do quadro depressivo, frequência e duração do afastamento.

**Conclusão:**

O afastamento laboral por transtornos mentais esteve associado à maior utilização de psicofármacos. O grupo de servidores identificado pode ser alvo prioritário de ações de promoção, prevenção e recuperação da saúde na instituição.

## INTRODUÇÃO

Os transtornos mentais (TM) representam, em sua maioria, condições prevalentes e incapacitantes, com baixas taxas de mortalidade. Dados do *Global Burden of Disease* (GBD) de 2017 mostram que os transtornos depressivos e transtornos de ansiedade, são responsáveis por elevada carga de TM, principalmente em pessoas em idade ativa. ^( 1 )^ No Brasil, esses transtornos se destacam pela maior carga de doença (35,0% para transtornos depressivos e 28,0% para transtornos de ansiedade), com maior contribuição para os anos vividos com incapacidade. ^( 2 )^

Um estudo japonês demonstrou que os TM eram a causa mais comum de afastamento laboral durante 30 dias ou mais entre trabalhadores de 20 a 59 anos. ^( 3 )^

Os TM representam 15,4% dos encargos totais de doença para as economias de mercado devido ao tempo de trabalho perdido ^( 4 )^ (somatório de horas semanais), por ausência do trabalho por razão pessoal, para acompanhar algum familiar doente (absenteísmo) ou pela redução de desempenho no trabalho relacionada à saúde (presenteísmo). ^( 4 )^

No Brasil, a prevalência de absenteísmo devido ao TM (causa principal) entre servidores públicos é de aproximadamente 25%. ^( 5 - 7 )^ Contudo, a prevalência varia de acordo com o diagnóstico e setores ocupacionais avaliados e com os aspectos metodológicos dos estudos. ^( 8 - 10 )^ Fatores prognósticos ou positivamente associados ao absenteísmo devido aos TM comuns incluem episódio anterior de adoecimento mental, maior gravidade da doença, registro prévio de afastamento, comorbidade, elevada carga, controle e demanda de trabalho, sexo feminino, baixa escolaridade, tabagismo e autoavaliação de saúde geral ruim. Trabalhadores mais jovens, com sintomas de menor gravidade, sem registro de afastamento anterior e com expectativas positivas sobre o afastamento e a retomada das atividades laborais retornam mais rapidamente ao trabalho. ^( 8 )^

A intervenção terapêutica ativa e sustentada dos transtornos psiquiátricos pode minimizar a ausência do trabalho. ^( 11 , 12 )^ O uso de psicofármacos está presente na vida diária de boa parcela da população, ^( 13 )^ incluindo a dos trabalhadores. ^( 9 , 11 , 12 )^ No entanto, estudos que avaliam a utilização de psicofármacos e o absenteísmo entre servidores públicos são escassos. A maioria refere-se aos trabalhadores da população geral ou a outros subgrupos específicos. ^( 8 )^

Estudo com 181 trabalhadores afastados do trabalho por causa psiquiátrica verificou que a totalidade dos casos recebeu tratamento psicofarmacológico relativamente adequado, habitualmente polifarmácia com até três princípios ativos, sendo mais comuns os antidepressivos inibidores seletivos e não seletivos da recaptação de serotonina, seguidos de ansiolíticos. ^( 11 )^ Por outro lado, uma coorte sueca demonstrou subutilização de antidepressivos por trabalhadores antes e após receberem pensão por incapacidade temporária ou permanente, ^( 14 )^ enquanto no Japão o não uso de antidepressivos foi superior entre trabalhadores com maior tempo de afastamento devido ao TM. ^( 12 )^ No Brasil, o Estudo Longitudinal de Saúde do Adulto (ELSA Brasil) mostrou que benzodiazepínicos e antidepressivos são comumente utilizados por funcionários e docentes universitários, mas sem investigar sua associação com o afastamento laboral. ^( 15 , 16 )^

A investigação acerca do tratamento dos TM que afetam os trabalhadores e sua associação com o tempo de afastamento laboral deve contribuir para o planejamento de ações de promoção à saúde mental do trabalhador e do manejo terapêutico dos TM.

## OBJETIVO

Descrever a utilização de psicofármacos por servidores públicos com registro de absenteísmo por transtornos mentais e analisar sua associação com a duração do afastamento do trabalho.

## MÉTODOS

### Desenho do estudo e período

Estudo transversal realizado a partir de dados secundários de servidores públicos federais em afastamento laboral por TM, no período de 1 ^o^ de janeiro a 31 de dezembro de 2017.

### População: critérios de inclusão e exclusão

A população do estudo consistiu em servidores públicos federais ativos, técnico-administrativos ou docentes da Universidade Federal de Minas Gerais (UFMG), em Belo Horizonte (MG). Os critérios de inclusão foram: registro de pelo menos um afastamento do trabalho por TM (capítulo V, grupo F, da Classificação Internacional de Doenças, 10ª Revisão – CID-10) ^( 17 )^ submetidos à perícia médica (dados de acesso restrito) no Departamento de Atenção à Saúde do Trabalhador (DAST) da UFMG (https://www.ufmg.br/prorh/dast/). Trabalhadores com registro de afastamento por TM não submetidos à perícia médica foram excluídos do estudo. Após aplicação dos critérios de exclusão, 202 servidores (equivalente a 2,8% dos servidores ativos, março de 2017) compuseram a amostra.

### Coleta de dados e variáveis

A coleta de dados foi realizada entre julho e outubro de 2018. Todas as fontes de dados utilizadas eram de acesso restrito. Os dados foram coletados dos prontuários médicos físicos de servidores submetidos à perícia no DAST e dos registros do Departamento de Administração de Pessoal (DAP). Nos prontuários, foram verificados: motivo de afastamento de acordo com CID F (causa principal do afastamento; CID principal e CID secundária, quando disponível); número e tempo (dias) de afastamento por CID F; e medicamentos (psicofármacos e outras classes) utilizados pelos servidores durante o afastamento (terceiro e quarto níveis da *Anatomical Therapeutic Chemical* (ATC) *Classification System* . Nos registros do DAP, foram buscadas variáveis demográficas e relacionadas ao trabalho (sexo, idade – calculada em junho de 2018 –, estado civil, escolaridade, cargo, nível do cargo, tempo de ocupação na instituição e tempo de trabalho). O nível do cargo foi classificado como de apoio (escolaridade exigida: ensino fundamental), intermediário (escolaridade exigida: ensino médio ou técnico nível médio) e superior (escolaridade exigida: ensino superior).

Foi realizado um estudo piloto para uniformização dos instrumentos de pesquisa e dos procedimentos da coleta de dados. Todos os dados foram tabulados em planilhas do software Excel, versão 2010 (Microsoft Corp., United States).

### Análise de dados

Os dados foram analisados por meio das frequências absolutas e relativas e medidas de tendência central e de dispersão. O coeficiente de correlação Rô de Spearman foi utilizado para verificar a correlação entre idade do servidor e tempo de afastamento. O teste não paramétrico de Kruskal-Wallis foi empregado para avaliar a correlação entre o uso de psicofármacos e o tempo de afastamento e o teste de Mann-Whitney, para correlação entre o uso de antidepressivo e o tempo de afastamento.

Técnicas estatísticas multivariadas de caráter exploratório (análise fatorial e análise de *cluster* ou conglomerados) foram utilizadas para verificar a associação entre as características profissionais e o perfil de adoecimento do servidor. Resumidamente, a análise de *cluster* consiste no agrupamento de n indivíduos (conglomerados) com base em características conhecidas sobre a forma de p variáveis. Indivíduos de um mesmo grupo ou conglomerado devem ser semelhantes entre si em relação às características que neles foram medidas, e os elementos em grupos diferentes devem ser heterogêneos em relação a essas mesmas características. ^( 18 )^ A análise fatorial (passo que precede a análise de *cluster* ) é realizada para eliminar o efeito de correlação observado entre variáveis. Variáveis correlacionadas (exemplo: tempo de trabalho e idade do servidor) são agrupadas, e novas variáveis são formadas (exemplo: variáveis funcionais) e utilizadas na análise de *cluster* . Utilizou-se o método hierárquico aglomerativo para a geração das sementes iniciais (centroides) e, em seguida, o método de K-médias, para os agrupamentos finais. Com base nos testes de Kruskal-Wallis, foram realizadas comparações entre as proporções de respostas às categorias das variáveis que compuseram os conglomerados obtidos durante a análise de *cluster.* ^( 18 )^

Todas as análises foram realizadas no software SPSS, versão 24.0.

### Aspectos éticos

O projeto de pesquisa foi aprovado pelo Comitê de Ética e Pesquisa envolvendo seres humanos da UFMG (parecer 2650149, CAAE: 86430018.6.0000.5149). Não foram realizadas entrevistas com os participantes, e as informações foram coletadas exclusivamente a partir de dados secundários. Por esse motivo, não foi necessária a apresentação do Termo de Consentimento Livre e Esclarecido (TCLE) aos participantes da pesquisa. Todo o estudo foi conduzido de acordo com a resolução 466/12, que regulamenta a pesquisa envolvendo seres humanos.

## RESULTADOS

### Características da população

Do total de participantes do estudo (n=202), 77,2% eram do sexo feminino, 70,3% tinham curso superior ou pós-graduação, e 41,6% eram casados. Aproximadamente 70% tinham 40 anos ou mais (média de idade 46,5 anos; variando de 24,7 a 67,5 anos) ( Tabela 1 ).

**Tabela 1:** Características dos servidores em afastamento laboral por transtornos mentais em uso de psicofármacos

Variáveis demográficas e relacionadas ao trabalho	n (%)
Sexo	
Feminino	156 (77,2)
Masculino	46 (22,8)
Faixa etária, anos	
20-30	12 (5,9)
30-40	44 (21,8)
40-50	66 (32,7)
50-60	65 (32,2)
60-70	15 (7,4)
Escolaridade	
Até ensino fundamental	5 (2,5)
Ensino médio	55 (27,2)
Ensino superior	40 (19,8)
Especialização	55 (27,2)
Mestrado	23 (11,4)
Doutorado	24 (11,9)
Estado civil	
Casado	84 (41,6)
Divorciado	25 (12,4)
Separado judicialmente	13 (6,4)
Solteiro	79 (39,1)
Viúvo	1 (0,5)
Nível do cargo*	
Nível apoio	5 (2,5)
Nível intermediário	130 (64,4)
Nível superior	49 (24,3)
Professor	17 (8,4)
Não informado	1 (0,5)

* nível do cargo: apoio (escolaridade exigida: ensino fundamental); intermediário (escolaridade exigida: ensino médio ou técnico nível médio); nível superior (escolaridade exigida: ensino superior).

Grande parte dos servidores tinha escolaridade superior à exigida para o cargo: 57,0% (74/130) dos servidores de nível intermediário eram graduados ou pós-graduados.

O tempo médio de trabalho foi de 15,7 anos (desvio-padrão – DP – de 9,8), sendo que 44% trabalhavam na UFMG há mais de 16 anos. O tempo médio de trabalho no serviço público foi de 16,6 anos (DP de 10,09).

### Afastamentos

Foram registrados 403 afastamentos, correspondendo a 10.698 dias de afastamento (média de 53 dias de afastamento/servidor) ( Tabela 2 ). Um quarto dos servidores se afastou por 12 dias ou menos, e outros 25% se afastaram por mais de 70 dias. O período de afastamento máximo foi de 420 dias. Cerca de 50% dos servidores registraram apenas um afastamento no período pesquisado.

**Tabela 2:** Distribuição do número de afastamentos e número de servidores usuários de psicofármacos por agrupamento de Classificação Internacional de Doenças

CID agrupadas	Afastamentos n (%)	Servidores n (%)
F00-F09 Transtornos mentais orgânicos, inclusive os sintomáticos	1 (0,2)	1 (0,5)
F10-F19 Transtornos mentais e comportamentais devidos ao uso de substância psicoativa	15 (3,7)	7 (3,5)
F20-F29 Esquizofrenia, transtornos esquizotípicos e transtornos delirantes	24 (6,0)	9 (4,5)
F30-F39 Transtornos do humor (afetivos)	187 (46,4)	101 (50,0)
F40-F48 Transtornos neuróticos, transtornos relacionados com o estresse e transtornos somatoformes	154 (38,2)	108 (53,5)
F50-F59 Síndromes comportamentais associadas a disfunções fisiológicas e a fatores físicos	3 (0,7)	1 (0,5)
F60-F69 Transtornos da personalidade e do comportamento do adulto	16 (4,0)	9 (4,5)
F70-F79 Retardo mental	1 (0,2)	1 (0,5)
Outro CID	1 (0,2)	1 (0,5)
Não informado	1 (0,2)	1 (0,5)
Total	403 (100,0)	202*

* corresponde ao número total de servidores e não à soma real da coluna, uma vez que o mesmo servidor teve diagnósticos em mais de um agrupamento da CID-10. CID: Classificação Internacional de Doenças.

Os transtornos de humor (afetivos) foram os mais frequentes, sendo responsáveis por 46,4% dos afastamentos e diagnóstico principal em metade dos servidores. Destacaram-se também os transtornos neuróticos, transtornos relacionados ao estresse e os somatoformes, sendo o diagnóstico principal em 53,5% dos servidores e responsável por 38,2% dos afastamentos ( Tabela 2 ). Esses TM também foram os principais quando consideradas as CID secundárias para estabelecimento do diagnóstico.

### Medicamentos

Um total de 187 (92,6%) servidores fazia uso de pelo menos um medicamento. Foram registrados 140 medicamentos distintos, sendo 14,3% antidepressivos utilizados por 82,9% dos servidores ( Tabela 3 ). Dentre os antidepressivos, destaca-se o uso de “inibidores seletivos da recaptação de serotonina” (N06AB), de “outros antidepressivos” (N06AX) e de “inibidores não seletivos da recaptação de monoamina” (N06AA), utilizados por 62,6%, 58,1% e 9,7% dos servidores, respectivamente. Já entre os antipsicóticos, destaca-se o uso de “diazepinas, oxazepinas, tiazepinas e oxepinas” (N05AH), de “outros antipsicóticos” (N05AX) e de “lítio” (N05AN), utilizados por 41,3%, 39,7% e 30,2% dos servidores, respectivamente. Dentre os ansiolíticos, foram utilizados “derivados de benzodiazepínicos” (N05BA) por 97,7% dos servidores e “derivados de azaspirodecanediona” por 4,7% dos servidores.

**Tabela 3:** Distribuição do número de psicofármacos utilizados segundo o *Anatomical Therapeutic Chemical Classification System* , terceiro nível, e número de servidores em afastamento laboral por transtorno mental

Psicofármacos	Medicamentos distintos n (%)	Servidores n (%)
Psicoestimulantes, agentes utilizados para TDAH e nootrópicos (N06B)	1 (0,7)	1 (0,5)
Opioides (N02A)	1 (0,7)	2 (1,1)
Ansiolíticos (N05B)	7 (5,0)	43 (23,0)
Hipnóticos e sedativos (N05C)	6 (4,3)	46 (24,6)
Antipsicóticos (N05A)	13 (9,3)	63 (33,7)
Antiepilépticos (N03A)	10 (7,1)	80 (42,8)
Antidepressivos (N06A)	20 (14,3)	155 (82,9)
Outros medicamentos (não psicofármacos)	82 (58,6)	107 (57,2)
Total	140 (100)	187 (100)*

* corresponde ao número total de servidores, e não à soma da coluna, uma vez que um mesmo servidor utilizou medicamentos em mais de um grupo de classificação. TDAH: transtorno do *deficit* de atenção com hiperatividade.

A mediana do tempo de afastamento de usuários que usam antidepressivos foi maior (mediana=30 dias) do que aqueles que não utilizaram (mediana=15 dias); teste de Mann-Whitney, valor de p=0,001.

Observou-se que 86% (n=174) dos servidores afastados faziam uso de algum psicofármaco, sendo que 69% deles utilizaram dois ou mais psicofármacos concomitantemente. O número médio de psicofármacos por servidor foi de 2,28 (DP de 1,49).

Foram observadas diferenças significativas nos valores centrais de dias de afastamentos por número de psicofármacos. Considerando as comparações múltiplas, o tempo de afastamento entre aqueles que não utilizam psicofármacos difere entre os que utilizaram dois (valor de p=0,004), três (valor de p<0,001), quatro ou mais psicofármacos (valor de p<0,0001) ( Figura 1 ).

**Figura 1:** Dias de afastamentos, por uso de psicofármacos

° *outlier* suave; * *outlier* extremo.

Não se observou correlação significativa entre o número de dias de afastamentos e idade dos servidores (Rô de Spearman de -0,026; valor de p=0,715).

### Análise de *cluster*

A análise resultou na obtenção de dois, três, quatro e cinco conglomerados. Os melhores arranjos resultaram na construção de três conglomerados, que obteve maior valor de R ^2^ (0,53), explicando 53% da variabilidade dos dados (α=0,05) ( Tabela 4 ).

**Tabela 4:** Características dos conglomerados de servidores em afastamento laboral por transtornos mentais em uso de psicofármacos

Variáveis	Total da amostra	Conglomerado			Valor de p
		1	2	3	
Uso de antidepressivo	77	71	70	96	<0,001
Diagnóstico de depressão ^*^	49	44	34	76	<0,001
Sexo					
Feminino	157 (78)	59 (79)	59 (78)	39 (76)	0,08
Masculino	45 (22)	16 (21)	17 (22)	12 (24)	
Nível do cargo ^†^					
Apoio	5 (2,5)	4 (5)	0 (0)	1 (2)	0,33
Intermediário	130 (64)	46 (61)	51 (67)	33 (65)	
Superior	49 (24)	16 (21)	21 (28)	12 (24)	
Professor	17 (8)	9 (12)	3 (4)	5 (10)	
Estado civil					
Casado	84 (42)	38 (51)	32 (42)	14 (28)	0,148
Solteiro	79 (39)	18 (24)	35 (46)	26 (51)	
Outros	39 (19)	19 (25)	9 (12)	11 (22)	
Afastamentos ^‡^	2,0±1,4	1,3±0,6	1,5±0,7	3,8±1,4	<0,001
Total de psicofármacos ^‡^	2,3±1,5	1,73±1,2	1,8±1,2	3,9±1,1	<0,001
Tempo de trabalho na UFMG, anos ^§^	15,7±9,8	24,0±6,8	8,0±4,8	15,0±9,3	<0,001
Idade do servidor, anos ^§^	46,4±9,9	54,4±5,5	38,6±6,8	46,3±9,5	<0,001
Total de dias de afastamentos ^‡^	53,0±67,2	25,9±24,7	28,3±30,7	129,6±91,4	<0,001

Resultados expressos como %, n (%) ou média±desvio-padrão. * compreende CID F31, F32, F33; ^†^ nível do cargo: apoio (escolaridade exigida: ensino fundamental); intermediário (escolaridade exigida: ensino médio ou técnico nível médio); superior (escolaridade exigida: ensino superior); ^‡^ compreende as “variáveis de afastamento”; ^§^ compreende as “variáveis funcionais”. UFMG: Universidade Federal de Minas Gerais.

No conglomerado 1 (n=75; 37,1%) e no 2 (n=76; 37,6%), estavam os servidores que se afastaram, em média, de 25 a 28 dias e menos de duas vezes no período. Aproximadamente 70% dos servidores dos dois grupos utilizavam antidepressivo. No conglomerado 1, 44% desses servidores tinham diagnóstico de depressão, e 34% com esse diagnóstico foram registrados no conglomerado 2. Os servidores utilizaram menos de dois psicofármacos concomitantemente (média de 1,7 psicofármaco). A diferença entre esses dois conglomerados está na idade dos servidores e no tempo de trabalho (média de idade de 54 anos e tempo médio de trabalho de 24 anos para o conglomerado 1 *versus* média de idade 38 anos e tempo médio de trabalho 8 anos para o conglomerado 2).

No conglomerado 3 (n=51; 25,4%), estavam os servidores que se afastaram, em média, 130 dias e aproximadamente quatro vezes em 2017. Quase a totalidade dos servidores (96%) utilizava antidepressivo, sendo que 76% destes tiveram diagnóstico de depressão. Os servidores utilizaram, em média, 3,86 psicofármacos concomitantemente. Possuíam média de idade de 46 anos e trabalhavam na universidade há, aproximadamente, 15 anos, em média.

Os três conglomerados não diferiram entre si quanto ao sexo, estado civil ou cargo dos servidores.

## DISCUSSÃO

O presente estudo avaliou a utilização de psicofármacos por servidores em afastamento laboral por TM e verificou sua associação com a duração dos afastamentos. A duração dos afastamentos esteve relacionada ao uso de psicofármacos, aumentando com o maior número de medicamentos utilizados. Esse achado difere dos resultados de outros autores que observaram que o tratamento farmacológico inadequado (não uso de psicofármacos ou subutilização de antidepressivos) está associado com o maior tempo de afastamento por TM ^( 12 )^ e com recebimento de pensão por incapacidade temporária ou permanente. ^( 14 )^

No estudo, os antidepressivos, os antiepilépticos e os antipsicóticos foram identificados como as principais classes de psicofármacos utilizadas, compatíveis com os diagnósticos F30-39 (transtornos do humor – afetivos) e F40-48 (transtornos neuróticos, transtornos relacionados com o estresse e somatoformes) registrados em aproximadamente 85% dos afastamentos. O uso concomitante de dois ou mais psicofármacos esteve presente em grande parte da população (69%), o que é definido por alguns autores como “polifarmácia psicotrópica ^”^ . ^( 19 )^ Embora a monoterapia deva ser a prática recomendada, várias doenças psiquiátricas requerem o uso de múltiplos medicamentos. ^( 20 )^ A polifarmácia pode ter sido necessária para o tratamento de casos de transtornos de humor associados aos transtornos de ansiedade observados em nosso estudo – e não necessariamente indicativa do uso excessivo do medicamento. Por outro lado, estudo do ELSA-Brasil sugeriu utilização inadequada de psicofármacos com subutilização de antidepressivos e superutilização de benzodiazepínicos por trabalhadores de instituições universitárias brasileiras. ^( 15 , 16 )^ Diferenças metodológicas e analíticas entre o nosso estudo e do ELSA-Brasil impossibilitam a comparação direta dos resultados. Por exemplo, enquanto esse último utilizou o autorrelato de todos os participantes (funcionários e docentes), o que amplia a detecção do uso de psicofármacos (e eventual inadequação), o nosso estudo analisou uma parcela relativamente selecionada de servidores periciados com registro secundário do uso de medicamentos, dificultando a mensuração de psicofármacos entre servidores com TM leves ou moderados que não geram afastamento do trabalho.

O uso de antidepressivos apresentou relação com a duração dos afastamentos e direcionou o agrupamento da população do estudo em três conglomerados. Os grupos compartilham a utilização de antidepressivos, que se difere em magnitude, e algumas características demográficas e relacionadas ao trabalho. Observamos um grupo minoritário (conglomerado 3) relativamente jovem e com tempo de trabalho intermediário comparativamente aos outros dois grupos, diferenciando-se na proporção de uso de antidepressivos, diagnóstico de depressão, frequência e duração do afastamento. O uso concomitante de quase quatro psicofármacos nesse grupo parece refletir o quadro depressivo grave desse grupo de servidores.

Aproximadamente 70% dos servidores afastados tinham 40 anos ou mais de idade. A mesma faixa etária de servidores afastados por TM foi observada em Alagoas ^( 21 )^ e Santa Catarina. ^( 6 )^ Outro estudo realizado em Santa Catarina entre 2010 a 2013 com 8.765 servidores afastados por TM ^( 22 )^ mostrou correlações fracas entre a idade dos servidores e o número de dias concedidos para licença para tratamento de saúde. Resultado semelhante foi encontrado no Piauí. ^( 23 )^ Em nossa pesquisa, os grupos se diferiram em relação à idade. Contudo, o conglomerado 3, que registrou maior utilização de psicofármacos (e antidepressivos), frequência e duração do afastamento, apresentou idade intermediária. De forma semelhante, uma coorte realizada na Dinamarca mostrou aumento no risco de afastamento por TM em trabalhadores em uso de antidepressivos com 25 a 54 anos, comparados aos grupos etários de 55 a 64 e 18 a 24 anos. ^( 9 )^ Em outros estudos, o afastamento laboral por TM foi mais frequente e longo em grupos etários mais velhos. ^( 8 , 10 , 24 , 25 )^

Não observamos diferenças de sexo, estado civil ou cargo ocupado pelos servidores. Alguns estudos mostram maior prevalência e incidência de TM em mulheres, ^( 8 , 10 , 24 , 25 )^ sugerindo que episódios depressivos estão associados à influência de pressões sociais, estresse crônico e baixo nível de satisfação associados ao desempenho de papéis tradicionalmente femininos, como cuidados com casa e filhos. ^( 26 )^ Apesar de os servidores, em sua maioria, serem do sexo feminino, essa característica parece não ser determinante para o consumo de psicofármacos (antidepressivos) na população estudada. Do mesmo modo, o cargo ocupado pelos servidores (em grande parte de nível intermediário) não definiu a formação dos grupos no estudo.

De forma inédita, este estudo avaliou a utilização de psicofármacos por servidores e sua associação com o afastamento do trabalho por TM em uma importante instituição de ensino superior brasileira. Algumas limitações devem ser, contudo, pontuadas. Casos menos graves de TM que geram afastamentos de curta duração podem ter sido subnotificados, decorrentes de situações pontuais em que servidores não comunicaram formalmente a ausência no trabalho ao DAST, mas sim à sua chefia direta. Do mesmo modo, o subregistro pode ocorrer quando o servidor com TM não se afasta do trabalho, por receio da exposição e da sujeição a atos preconceituosos no ambiente laboral. Ademais, pode ter ocorrido subregistro dos psicofármacos em prontuários, ocasionado pelo relato incompleto do servidor no momento da perícia, relacionado ao esquecimento ou, até mesmo, ao constrangimento associado aos TM e/ou ao uso de psicofármacos, que revelam estigma em relação à doença e ao tratamento. Ressaltamos ainda que, devido à complexidade da doença e do tratamento farmacológico dos TM, focamos em uma avaliação quantitativa da utilização do psicofármaco. O emprego de dados secundários limita o acesso a informações sobre uso de outros medicamentos e ao modo como são efetivamente utilizados, desfavorecendo uma abordagem qualitativa da adequação do uso do medicamento.

A importância dos TM tem se revelado na geração de afastamentos laborais em servidores públicos no Brasil. ^( 5 - 7 )^ Na instituição avaliada, os TM e comportamentais foram responsáveis por mais de dez mil dias de afastamento por ano entre 2011 e 2017, ^( 27 )^ ocupando a primeira posição no *ranking* do número de dias de afastamento. A condução de outras investigações, especialmente de estudos longitudinais que permitam investigar o uso qualificado de psicofármacos na trajetória do afastamento laboral, é fundamental para a evolução do conhecimento entre trabalhadores de instituições públicas de ensino superior no Brasil.

## CONCLUSÃO

O afastamento laboral por transtornos mentais esteve associado a maior utilização de psicofármacos. O padrão de utilização de antidepressivos discriminou um de três grupos de servidores, caracterizado pela maior duração de afastamento por transtornos mentais, idade e tempo de trabalho intermediário e maior uso de psicofármacos (polifarmácia). Essa análise exploratória inédita entre trabalhadores de instituições públicas de ensino superior no Brasil pode auxiliar no estabelecimento de estratégias de ações de promoção, prevenção e recuperação à saúde do trabalhador.
